# Efficacy of Three Different Methods in the Retreatment of Root Canals Filled with Resilon/Epiphany SE

**Published:** 2010-11-15

**Authors:** Hasan Ramzi, Noushin Shokouhinejad, Mohammad Ali Saghiri, Ardavan Samieefard

**Affiliations:** 1. Department of Endodontics, Dental Research Center/School of Dentistry, Tehran University of Medical Sciences, Tehran, Iran.; 2. Department of Endodontics, Dental Research Center/School of Dentistry, Tehran University of Medical Sciences, and Iranian Center for Endodontic Research, Tehran, Iran.; 3. Department of Dental Material, Dental School, Azad University/Kamal Asgar Research Center (KARC), Tehran, Iran.; 4. Department of Endodontics, School of Dentistry, Tehran University of Medical Sciences, Tehran, Iran.

**Keywords:** Chloroform, Endosolv R, Resilon, Epiphany, Retreatment, Self-etch

## Abstract

**INTRODUCTION:**

The purpose of this study was to evaluate the efficacy of three methods in removal of Resilon/new Epiphany self-etch (SE) soft resin endodontic obturation system.

**MATERIALS AND METHODS:**

Thirty extracted single rooted human teeth were prepared for endodontic treatment and obturated with Resilon/Epiphany SE. The roots were randomly divided into three groups; group 1 roots were retreated using Mtwo R/Mtwo files; group 2 were retreated using Mtwo R/Mtwo accompanied with chloroform; and group 3 were retreated using Mtwo R/Mtwo accompanied with Endosolv R. The cleanliness of canal walls was determined using scanning electron microscopy. Data were analyzed using ANOVA and LSD tests.

**RESULTS:**

Endosolv R combined with rotary files was more efficient in material removal compared to chloroform combined with rotary files and rotary files alone (P<0.05). Also, chloroform combined with rotary file was more efficient than rotary file alone in removing filling material from the root canals. Significant difference was found within group 1 between the coronal third compared to the middle and apical thirds (P<0.05). In group 2, there were more material remnants in the apical third (P<0.05). In group 3, there was no significant difference between the three segments of the root canals (P>0.05).

**CONCLUSION:**

All techniques left filling material remnants and debris on the root canal walls. Endosolv R combined with rotary files most effectively removed filling materials from the root canals, especially in the apical third.

## INTRODUCTION

Subsequent to root canal treatment, retreatment might be necessary due to persistent infection or re-infection of the root canal system [1]. The main objective of nonsurgical root canal retreatment is to re-constitute healthy periapical tissues [2]. Therefore, the complete removal of root filling material is necessary for retreatment. Different techniques have been advocated for removing filling materials. These include hand or rotary instrumentation either alone or combined with heat or solvents ultrasonic instruments, laser and paper point, with chemicals [3].

Resilon (Resilon Research LLC, Madison, CT) is a thermoplastic synthetic polymer-based root canal filling material containing bioactive glass and radiopaque fillers [4] and is used with a resin-base sealer (Epiphany Pentron Clinical Technologies, Wallingford, CT). The first generation of Epiphany obturation system consisted of a core material (Resilon), a dual-curable resin-based sealer (Epiphany), and a self-etching primer [4][5]. A new Epiphany self-etch (SE) soft resin endodontic obturation system (Pentron Clinical Technologies, Wallingford, CT), has been marketed consisting of two components: Epiphany SE sealer and Resilon. The Resilon can be removed with files or softened with heat or solvents. Chloroform is the most common solvent in root canal retreatment. It is toxic in contact with periradicular tissues; it is also hepatotoxic, and nephrotoxic [6]. Furthermore, chloroform may change the chemical composition of the dentin surface thereby decreasing bond strength of bonded materials [7][8][9]. In addition, the elimination of the resin sealer tags of Resilon/Epiphany (Pentron Clinical Technologies, LLC, Wallingford, CT) that have penetrated deep into the dentinal tubules might be difficult for chloroform [4]. Therefore, the use of a resin solvent such as Endosolv R (Septodont, Paris, France), designed for softening resin-based pastes [10], is likely to result in better resin sealer removal [11]. However, the efficacy of this solvent to remove Resilon/Epiphany system has not been thoroughly investigated. There is only one study comparing the effect of Endosolv R and chloroform on the bond strength of Resilon/Epiphany SE to intracanal dentin during retreatment [9].

Nickel-titanium rotary (NiTi) files have also been used for removing the resin-based root canal fillings during endodontic retreatments [12][13][14][15][16][17]. Mtwo R (VDW, Munich, Germany) file is a NiTi rotary instrument which has been designed for retreatment [13].

Different studies have evaluated the efficacy of various techniques for Resilon/Epiphany removal from the root canal [12][13][14][15][16][17]; however, studies have not evaluated the efficacy of different retreatment obturation techniques and materials for example new Epiphany SE soft resin as an endodontic obturation system. In addition, there are no published articles discussing the efficacy of resinous solvent (Endosolv R) in retreatment of root canals filled with Resilon/Epiphany SE. The purpose of this study was to compare the efficacy of retreatment techniques using Mtwo R/Mtwo instruments either alone or combined with chloroform or Endosolv R in removing Resilon/epiphany SE root canal filling material.

## MATERIALS AND METHODS

Thirty single-rooted, extracted human teeth were selected and stored in 0.5% chloramines-T before use. The teeth were decoronated to a standardized root length of 13-15mm. The working length was established at 1mm short of the file’s emergence at the apical foramen.

The root canals were instrumented using Mtwo rotary instruments to an apical size of 35/04. Root canals were irrigated between each two instruments with 5mL of 2.5% NaOCl solution. After root canal preparation, the canals were irrigated with 5mL of 17% EDTA and then finally rinsed with 10mL of saline solution. The root canals were then dried with paper points and obturated with Resilon/Epiphany SE sealer according to the process outlined below. A Resilon master cone (size 35/02 taper) was coated with Epiphany SE sealer and placed gently within the root canal. The size 20/02 taper accessory Resilon cones were used for canal obturation by using the cold lateral compaction technique.

The quality of obturation was then confirmed radiographically. The excess Resilon was removed with a heated instrument and compacted with a cold plugger. The coronal surface of the obturation was light cured for 40 seconds according to the manufacturer’s instructions. The specimens were stored in an incubator at 37ºC and 100% humidity for 8 weeks. Then, the teeth were randomly divided into three groups (n=10).

***Group 1:*** Retreatment with rotary files Mtwo R/Mtwo

In this group obturating material was gradually removed with Mtwo R 25/05 and Mtwo R15/05 files, respectively, in a brushing action. After the working length was reached, the following conventional Mtwo files were used to remove the filling material: Mtwo 15/.05, 20/.06, 25/.06, 30/.05, 35/.04, and 40/.04.

***Group 2:*** Retreatment with rotary files, Mtwo R/Mtwo, and Chloroform 

The coronal portion of the filling material was removed with Gates-Glidden drills sizes 2 (Maillefer Dentsply, Ballaigues, Switzerland). Then, two or three drops of chloroform (Merck, Darmstadt, Germany) were introduced into each canal to soften the filling material. The remaining root canal filling material was removed with Mtwo R and Mtwo files in the same manner as group 1. Additional drops of chloroform were used to reach the working length. In the final step, the canal was filled with chloroform for 1 minute, and the solution was then absorbed and removed with paper points size 40/02. This process was repeated three times. In each specimen a total of 1mL of chloroform was used.

***Group 3:*** Retreatment with Mtwo R/Mtwo files and Endosolv R 

In this group, all retreatment procedures were performed in the same manner as group 2, except retreatment was performed with Endosolv R instead of chloroform. A total of 1mL of Endosolv R was used in each tooth.

The Mtwo and Mtwo R instruments were operated in an electric handpiece (X-Smart; Dentsply Maillefer, Ballaigues, Switzerland) at a constant speed of 300 rpm and torque recommended by the manufacturer. During the retreatment procedure each rotary instrument was used to retreat four canals.

During retreatment, the root canals were irrigated with 2.5% NaOCl. The criteria for completion of retreatment were the presence of smooth canal wall and free of visible debris outlined by a radiograph, no evident filling material on the files or paper points. After final instrumentation, the root canals were irrigated with 5mL of 17% EDTA for 1 minute and finally flushed with 10mL of saline solution. After that, the canals were dried with paper points and prepared for scanning electron microscopy (SEM) analysis.

All samples were subjected to the desiccant to reduce air humidity (removing air moisture). After that, two longitudinal grooves were prepared on the buccal and lingual surfaces of each root using a diamond disc without penetration into the canal. The roots were then split into two halves with a chisel. One half of each sample was randomly chosen, placed in 2% glutaraldehyde for 24 hours and then rinsed 3 times with sodium cacodylate buffered solution (pH 7.2). After incubation in osmium tetroxide for 1 hour, the samples were dehydrated with ascending concentrations of ethyl alcohol (30-100%), placed in desicators for 24 hours and mounted on a metallic stub. Each specimen was sputter coated with gold and examined under scanning electron microscope (LEO 440i, Oxford, UK) equipped with secondary electron detector. After taking SEM micrographs of each specimens segment (apical, middle, coronal) with ×500 magnification, the post processing step of digital subtraction of remaining material was performed by using a scientific programming package (MATLAB; Math works, Natick, MA). The amount of residual filling material and debris was evaluated according to the following criteria:

1. None to slight presence (0%-25%) of residual debris covering the dentinal surface;

2.Mild/Moderate presence (25%-50%) of residual debris on the surface;

3.Moderate/Severe presence (50%-75%) of residual debris

4.Severe or complete presence (75%-100%) is covered with residual debris.

No attempt was made to distinguish between filling material or sealer remnants [15].

ANOVA and Post Hoc tests (LSD) were used for analysis. The level of significance was set at P<0.05.

## RESULTS

**Figure 1:**
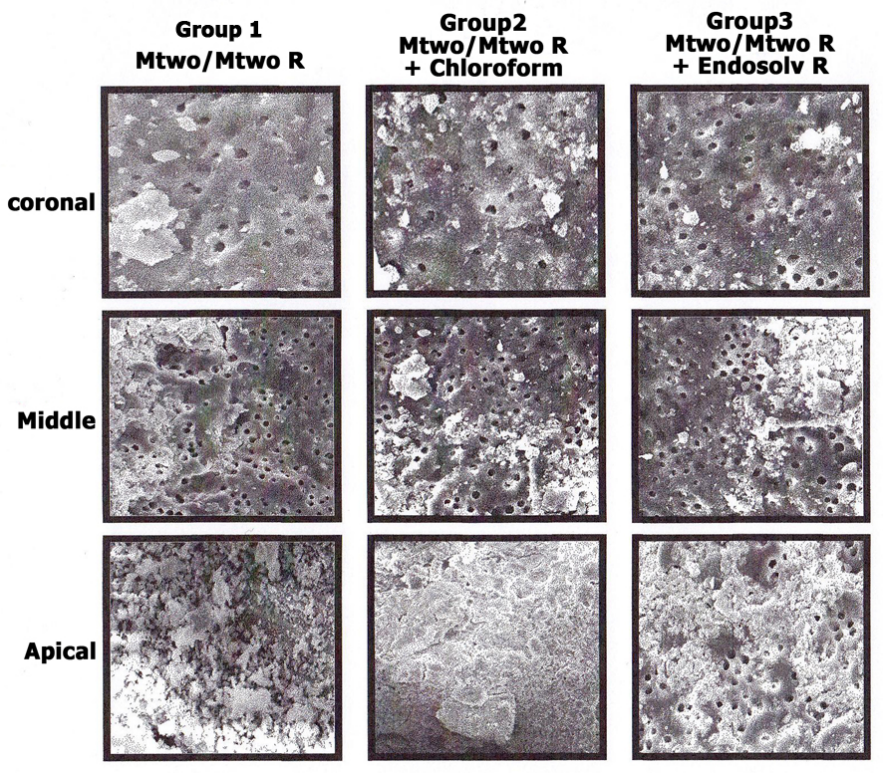
Selective SEM images of the coronal, middle, and apical areas of samples in each of three groups (original magnification 500×).

Residue of the root-filling materials was observed in all specimens regardless of the used techniques. The mean ratio of residual filling material on canal walls is shown in Table 1. Significant differences were found between three techniques regarding the residual debris (P<0.05). The group retreated using Mtwo R/Mtwo instruments combined with Endosolv R demonstrated the least residual debris all along the root canals (P<0.05). In the group using Mtwo R/Mtwo instrument combined with chloroform, less debris was found compared coronal, middle and apical segments, group 2 (Mtwo R/Mtwo with chloroform) demonstrated more filling remnants in the apical third compared with the middle and coronal third (P<0.05). In samples retreated with Mtwo R/Mtwo only (group 1) only the coronal segment demonstrated significantly greater mean score cleanliness compared with the apical and middle thirds (P<0.05), the difference between middle and apical was not significant. In group 3 (Endosolv R group), there was no statistically significant difference between apical, middle, and coronal thirds (P=0.891). Selective SEM images of the coronal, middle, and apical thirds of samples in each of the three experimental groups are shown in Figure 1.

**Table 1:** Mean scores (SD) of canal wall cleanliness for different retreatment groups at the coronal, middle, and apical portions

**Group **	**Coronal**	**Middle**	**Apical**	**Total**
**Mtwo R/Mtwo**	9.32(1.70)	14.19(3.95)	16.35(1.62)	13.29(3.93)
**Mtwo R/Mtwo** **+Chloroform**	6.17(1.08)	7.49(1.38)	11.32(1.00)	8.32(2.49)
**Mtwo R/Mtwo** **+Endosolv R**	3.89(2.29)	4.27(3.42)	4.50(2.77)	4.22(2.77)

## DISCUSSION

The success of endodontic retreatment is directly related to the complete removal of the obturation material from the root canal. In the present study, a self-etch/self-adhesive type sealer (Epiphany SE) was used as these methacrylate with the group retreated with Mtwo/Mtwo instruments (P<0.05). When comparing the resin-based sealers do not required several stages thereby saving time [18].

Previous studies have suggested greater canal space enlargement to reduce remnants of root canal filling material [19][20]. Therefore, retreatment procedure was performed with 1 size larger (Mtwo 40/04) than the file used in instrumentation before root canal filling (Mtwo 35/04). It has been suggested that wicking action is essential in removing residual filling material and sealer out of fins and aberrations of the root canal systems [21]. Therefore, for maximum removal of Resilon/Epiphany from the root canal system, the chemical flushing with chloroform and wicking procedures were performed in this study. Chloroform has been shown to be quite an effective solvent for Resilon/Epiphany [20][22]. Previous studies demonstrated that retreatment of Epiphany system was enhanced by the use of chloroform [13][20]. However, chloroform has potential of damage to periapical tissues, systemic toxicity, and imposes a health hazard to dental personnel through frequent chloroform vapor inhalation [23][24]. Furthermore, some investigations showed that chloroform has a negative effect on bond strength of bonding material to root canal dentin [8][9]. It has been stated that solvents may change the chemical composition of the dentin surface thereby altering bond strength [7]. These agents could solubilize the lipids in dentin [25][26]. These lipids may redeposit on the dentin surface as a waxy film that may interfere with development of resindentin bonds [8].

In the retreatment of Resilon/Epiphany, eradicating the resin sealer tags which have penetrated in dentinal tubules is difficult. Therefore, the use of a resin solvent such as Endosolv R may be useful [11]. Endosolv R was originally designed for softening hardened Resorcinol-formaldehyde resin [27].

In the present study, no technique was able to wholly eliminate the previous root filling; residual canal filling material was observed in all three groups, concurring with previous studies [12][13][16][28].

This study showed that during material removal, the use of Endosolv R combined with Mtwo R/Mtwo instruments resulted in significantly greater residual mater removal compared to mechanical instrumentation alone or mechanical instrumentation combined with chloroform. Also the use of chloroform combined with mechanical instrumentation was more efficient than mechanical instrumentation alone, which is in accordance with Hassanloo et al. [29].

In the present study, during material removal, canals in chloroform group tended to accumulate more debris apically. This is in agreement with Ezzie et al. [13]. Several investigations have shown that the highest residue value were recorded in the apical third of canal [28][30][31], which is in contrast with the findings of this study related to specimens that were retreated with Endosolv R combined mechanical instrumentation. In Endosolv R group there was no statistical significant difference in residual filling material left on canal walls between apical, middle and coronal thirds of the canal. The solubility of Resilon/Epiphany SE in Endosolv R may have ntributed to this difference. Therefore, employment of Endosolv R in retreatment of canals filled with Resilon/Epiphany SE system might improve the clinicians’ ability to reduce apical residue. Under the conditions of the present experimental study, Resilon/Epiphany SE obturation system was re-treatable with solvents and rotary files. All techniques left filling material within the root canal.

## CONCLUSION

A combined use of rotary files and Endosolv R may achieve the desired optimum results especially in apical third of the canal. Further investigations are recommended to evaluate the efficacy of this solvent on the clinical success of retreatment cases.
